# Hybrid deep learning model for accurate and efficient android malware detection using DBN-GRU

**DOI:** 10.1371/journal.pone.0310230

**Published:** 2025-05-19

**Authors:** Heena Kauser.Sk, Maria Anu.V

**Affiliations:** 1 Research scholar, Department of Computer Science & Engineering, Sathyabama Institute of Science and Technology, Chennai, Tamil Nadu, India; 2 Department of Computer Science and Engineering, Vellore Institute of Technology, Chennai, Tamil Nadu, India; OU: The Open University, UNITED KINGDOM OF GREAT BRITAIN AND NORTHERN IRELAND

## Abstract

The rapid growth of Android applications has led to an increase in security threats, while traditional detection methods struggle to combat advanced malware, such as polymorphic and metamorphic variants. To address these challenges, this study introduces a hybrid deep learning model (DBN-GRU) that integrates Deep Belief Networks (DBN) for static analysis and Gated Recurrent Units (GRU) for dynamic behavior modeling to enhance malware detection accuracy and efficiency. The model extracts static features (permissions, API calls, intent filters) and dynamic features (system calls, network activity, inter-process communication) from Android APKs, enabling a comprehensive analysis of application behavior.The proposed model was trained and tested on the Drebin dataset, which includes 129,013 applications (5,560 malware and 123,453 benign).Performance evaluation against NMLA-AMDCEF, MalVulDroid, and LinRegDroid demonstrated that DBN-GRU achieved 98.7% accuracy, 98.5% precision, 98.9% recall, and an AUC of 0.99, outperforming conventional models.In addition, it exhibits faster preprocessing, feature extraction, and malware classification times, making it suitable for real-time deployment.By bridging static and dynamic detection methodologies, the DBN-GRU enhances malware detection capabilities while reducing false positives and computational overhead.These findings confirm the applicability of the proposed model in real-world Android security applications, offering a scalable and high-performance malware detection solution.

## 1. Introduction

The Android operating system (OS) has become a dominant force in the mobile ecosystem, with over 70% of global devices currently operating on this platform [1].By 2025, approximately 5.2 billion smartphones and Internet of Things (IoT) devices are projected to be powered by Android [2].This rapid proliferation has enhanced global connectivity and mobile innovation but has also introduced significant security issues.The increasing volume of downloadable applications—exceeding 2.8 million on the Google Play Store—has made this platform a frequent target for cybercriminals.Between June 2018 and March 2022, over 746,000 malicious applications were identified on Android [3], posing serious threats to user privacy, financial data, and device integrity.

Traditional malware detection approaches, particularly those based on static signature matching, offer reliable protection against known threats but remain inadequate in detecting sophisticated variants, such as polymorphic, metamorphic, and zero-day malware [4,5].These evolving threats often bypass conventional defenses by modifying their structures or exploiting unknown vulnerabilities, emphasizing the need for more adaptable detection mechanisms.

To mitigate these limitations, both static and dynamic analysis techniques have been investigated.Static analysis evaluates the code of an application without execution, enabling low-cost preliminary assessments.In contrast, dynamic analysis observes runtime behavior in sandbox environments and is more effective against obfuscated or evasive malware.However, its high computational cost and unsuitability for real-time implementation on resource-constrained Android devices are critical drawbacks [6]. As Android malware continues to grow in complexity, there is a compelling need for intelligent, lightweight, and adaptive models that can perform effective detection in dynamic environments [7].

Machine learning (ML) has emerged as a promising approach that enables models to learn from large-scale datasets and generalize across variants.Nonetheless, conventional ML approaches, such as Support Vector Machines (SVM) [8], k-nearest neighbors (k-NN) [9], and regression-based classifiers [10], are limited by shallow architectures and fail to capture the deep, nonlinear relationships inherent in malware behavior.

To address these gaps, this study proposes a hybrid DBN-GRU-based detection model that combines the hierarchical feature abstraction capabilities of Deep Belief Networks (DBNs) [11] with the temporal sequence modeling strength of Gated Recurrent Units (GRUs). DBNs extract high-level static and dynamic features, whereas GRUs capture temporal dependencies and behavioral evolution over time.This integration enables comprehensive malware behavior modeling across both feature domains, thereby improving the detection accuracy and robustness against previously unseen or obfuscated malware variants.

This study was driven by the following specific objectives:

- To develop a hybrid DBN-GRU model capable of extracting deep semantic representations and modeling temporal behaviors for the accurate classification of Android malware across both static and dynamic feature spaces.
- To benchmark the proposed model against existing state-of-the-art approaches, including NMLA-AMDCEF [12], MalVulDroid [13], and LinRegDroid [14], we highlighted its comparative performance, strengths, and limitations.

The aim of overarching is to design a scalable and intelligent malware detection model that addresses the shortcomings of conventional methods while contributing to the development of resilient Android security solutions.The intended beneficiaries of this study include mobile security researchers, malware analysts, platform providers, and developers of Android security tools, who may adopt or extend this model in real-world security infrastructures.

The remainder of this paper is structured as follows: Section 2 presents a comprehensive review of existing Android malware detection techniques, identifies research gaps, and contextualizes the proposed work.Section 3 describes the methodology and architectural design of the DBN-GRU model, which integrates static and dynamic analyses.Section 4 outlines the experimental setup, datasets, and evaluation metrics, followed by the comparative results.Section 5 discusses the limitations and future directions of this study.Section 6 concludes with a summary of the contributions and recommendations for advancing Android malware detection research.

## 2. Literature survey

This section presents a comprehensive analysis of recent advancements in Android malware detection, focusing on traditional machine learning techniques, hybrid models, and deep learning applications aimed at enhancing detection accuracy. The review is structured to identify existing gaps and substantiate the development of the proposed hybrid DBN-GRU model.

### 2.1 Android malware threat landscape and detection challenges

The Android malware landscape has evolved considerably, thereby challenging existing detection methods.Android’s widespread adoption and open-source nature have made it a prime target for cyber threats, leading to a surge in the number of malicious applications[]. Advanced obfuscation techniques have transformed malware detection into a complex big data issue that traditional methods struggle to address effectively [15]. Machine learning algorithms often encounter computational challenges because of high-dimensional feature sets[]. Additionally, malware developers frequently alter features to maintain malicious functionality, diminishing the efficacy of handcrafted feature-based detection approaches [16]. The phenomenon of app collusion, where multiple applications collaborate to perform malicious activities, further complicates detection by necessitating multi-app analyses to uncover new security vulnerabilities.Other complexities, such as dynamic code loading and biases in experimental datasets, add to these challenges [17].

To mitigate these issues, researchers have turned to deep learning models for analyzing malware behavior. Integrating dynamic feature analysis with deep learning methods has enhanced detection capabilities against both known and novel malware variants [18]. However, the evolving nature of Android malware requires continuous innovation in detection methodologies to keep up with emerging threats.

### 2.2 Machine learning approaches for malware detection

Machine learning techniques have been extensively utilized for malware detection because of their capacity to learn patterns from large datasets and adapt to evolving threats.Traditional algorithms, including Support Vector Machines (SVM) [19], Naïve Bayes (NB), and Decision Trees, have demonstrated notable accuracy and efficiency. For example, an optimized Gradient Boosted Decision Tree model achieved a detection accuracy of 96.38% with a false positive rate of 0.004 on the EMBER dataset [20].

Deep learning techniques have further advanced malware detection by effectively capturing these complex patterns.Recent studies have highlighted the superior performance of Deep Neural Networks (DNN) in handling large-scale datasets and sophisticated malware variants [21].However, some studies suggest that high-performing malware detectors do not necessarily require the most complex machine learning models [22], indicating the importance of feature engineering and dataset quality.The concept of ‘malware analysis economics’ has emerged, focusing on maximizing detection accuracy while minimizing computational costs. Although machine learning approaches [23] have demonstrated high accuracy, challenges remain in feature selection, addressing dataset biases, and ensuring real-time performance on resource-constrained devices. Future research should focus on enhancing model interpretability, resilience against adversarial attacks, and computational efficiency [24].

### 2.3 Static, dynamic, and hybrid analysis techniques

Malware detection techniques are generally categorized into static, dynamic, and hybrid approaches, each with distinct advantages and limitations. Static analysis focuses on features such as API calls, code structures, and system library usage.Although effective against known malware, static analysis is vulnerable to obfuscation techniques [25].

Dynamic analysis observes malware behavior during execution in a controlled environment. It is particularly effective against metamorphic malware but may face limitations in runtime performance for certain edge cases [26]. The hybrid analysis combines static and dynamic approaches to leverage their respective strengths. For instance, the AM Detector system tags attack tree nodes based on static analysis and verifies runtime behaviors through dynamic analysis [27].

Another hybrid approach involves mining dynamic attributes to classify executables as benign or malicious [28]. Studies employing Hidden Markov Models (HMMs) with combined static and dynamic features have demonstrated superior detection rates, underscoring the need for hybrid models[].As malware continues to evolve, hybrid analysis techniques are gaining prominence because of their ability to overcome the limitations of individual methods [29].

### 2.4 Deep learning models for enhanced detection accuracy

Deep learning models, such as Convolutional Neural Networks (CNN) and Recurrent Neural Networks (RNN), have shown promising results in malware detection by automatically extracting hierarchical features from complex data [30,31]. These models outperform traditional machine learning approaches by capturing intricate patterns and effectively addressing obfuscation techniques.

Despite their accuracy, deep learning approaches are computationally intensive and require large training datasets, which pose challenges for resource-constrained mobile devices.Studies have emphasized the need for optimized architectures and techniques to balance detection performance with resource efficiency.Recent research highlights the limitations of single-mode analysis and the growing adoption of hybrid deep-learning models.These models integrate multiple feature types to enhance the detection resilience.For example, [32] proposed a neural network-based model that combined static and dynamic features to improve generalizability, whereas [33] developed a lightweight, interpretable model for malware classification.

Hybrid deep learning models leverage features such as permissions, API calls, and network behavior to improve the detection accuracy.The combination of DBN for feature extraction and GRU for temporal sequence analysis has demonstrated the potential to effectively capture complex patterns. This study proposes a novel DBN-GRU model that integrates these techniques to improve malware detection by extracting features and processing sequential patterns in a unified model.

### 2.5 Comparative analysis and research gaps

To further highlight the gaps in the literature, Table 1 provides a comparative analysis of existing techniques based on their detection accuracy, computational overhead, and adaptability.

**Table 1:** Comparative analysis of android malware detection techniques.

Author(s)	Approach	Model used	Accuracy (%)	False Positive Rate	Computational Overhead	Adaptability
**Kumar and Subbiah [5]**	Ensemble Learning	Shapley Ensemble Boosting and Bagging approach for zero-day malware detection	98.6%	1.2%	Moderate; ensemble methods increase complexity	High; effective against unknown malware variants
**Sanjaa and Chuluun [8]**	Machine Learning	Linear Support Vector Machine (SVM) for malware detection	96.5%	Not specified	Low; linear SVMs are computationally efficient	Moderate; may require retraining for new malware
**Rao et al. [9]**	Machine Learning	Improved K-Nearest Neighbors (KNN) model for malware detection and classification	94.7%	3.1%	Moderate; depends on dataset size and feature dimensions	High; adaptable with updated training data
**Alswaina and Elleithy [10]**	Hybrid Analysis	Combination of static and dynamic analysis for Android malware family classification	97.8%	2.0%	High; due to dual analysis methods	High; robust against various malware obfuscation techniques
**Khanna et al. [7]**	Feature Selection	BorutaShap algorithm for efficient Android malware detection	95.2%	2.5%	Moderate; feature selection reduces dimensionality	High; focuses on relevant features, improving adaptability
**Wang and Zong [16]**	Signature-Based	DroidGene: Detecting Android malware using its malicious gene	93.4%	4.0%	Low; signature-based methods are less resource-intensive	Low; limited effectiveness against new malware variants
**Aldhafferi [18]**	Machine Learning	Support Vector Regression for dynamic feature analysis in Android malware detection	92.8%	3.7%	Moderate; dynamic analysis increases computational load	High; effective in detecting behavior-based malware
**Zhao et al. [27]**	Hybrid Method	AM Detector: A hybrid method for Android malware detection	95.0%	2.8%	High; combines static and dynamic analysis	High; robust against various malware techniques
**Vidyarthi et al. [28]**	Hybrid Approach	Detection of malware using text mining and dynamic analysis	94.5%	3.0%	High; due to combined methodologies	High; effective against polymorphic malware
**Yang et al. [29]**	Hybrid Analysis	Text mining and machine learning for Android malware analysis	96.2%	2.3%	High; hybrid approaches increase computational load	High; effective against various malware types
**Ahmad [31]**	Deep Learning Model	Enhanced malware detection using deep learning for feature extraction and classification	97.0%	1.8%	High; deep learning models are resource-intensive	High; capable of adapting to new malware patterns
**Zhang et al. [32]**	Multimodal Analysis	MPDroid: A multimodal pre-training Android malware detection method with static and dynamic features	98.2%	1.5%	High; multimodal approaches require substantial resources	High; robust against sophisticated malware
**Palma et al. [33]**	Explainable AI	Explainable machine learning for malware detection on Android applications	96.8%	2.0%	High; explainable AI adds computational complexity	High; improves trust and adaptability in detection systems

This comparative study underscores the breadth of methodologies explored for Android malware detection, including traditional machine learning algorithms, advanced deep learning architectures, and integrated hybrid models.Ensemble-based approaches, such as the Shapley Ensemble Boosting and Bagging technique}, demonstrate notable accuracy and resilience against zero-day threats; however, they are associated with moderate computational demands owing to their model complexity. Linear SVM-based models}, while efficient and lightweight, may require frequent retraining to remain effective in the face of evolving malware behaviors. In contrast, hybrid techniques that fuse static and dynamic analyses enhance robustness against obfuscation and polymorphism, although they incur higher resource consumption.

This comparison emphasizes the critical need for detection models that strike an optimal balance between performance, computational overhead and adaptability. To address these challenges, the proposed DBN-GRU model integrates the hierarchical feature extraction capabilities of Deep Belief Networks (DBN) with the temporal learning strength of Gated Recurrent Units (GRU). This unified architecture is designed to enhance detection accuracy while preserving efficiency and adaptability, making it well suited for real-world Android malware detection scenarios.

## 3. Proposed model

A systematic approach to building the proposed Android malware detection model is presented in the Methodology section.This structured process covers all key phases, from data collection and preprocessing to feature extraction and selection, model architecture design, training and validation processes, and metrics used to evaluate the model performance.Each phase is specifically designed to guarantee the robustness and effectiveness of the detection system regarding the complexity of the static and dynamic analyses of Android applications.

**Fig 1:**
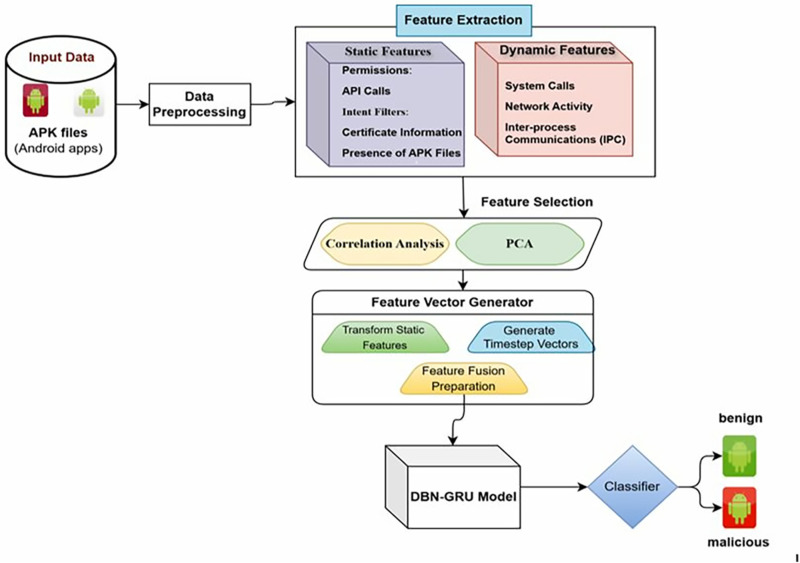
Overall Block diagram of the Proposed model.

The architecture of the Android malware detection model based on the hybrid DBN-GRU approach is presented in Fig 1.The input data contained Android APK files, which were obtained through data preprocessing, and their input was standardized for analysis.Specifically, feature extraction categorizes static features (e.g., permissions, API calls, intent filters, certificate information, and presence of APK files) and dynamic features (e.g., system calls, network activity, and inter-process communications).Once extracted, the feature selection class was applied to perform correlation analysisand Principal Component Analysis(PCA) to reduce dimensionality and extract the most relevant features.Static features are transformed into a feature vector generator, dynamic features are converted into time step-specific vectors, and both are fused into a unified feature vector.The DBN-GRU model acts as an input to the fused representation and uses static and dynamic analysis strengths for more robust malware detection.A classifier is then used to determine whether the application is benign or malicious based on the model’s output.

### Ethics statement

The study was conducted in accordance with ethical guidelines.

### 3.1 Data collection and preprocessing

#### 3.1.1 Data collection.

A comprehensive data collection strategy was employed to assemble a representative dataset comprising both benign and malicious Android applications. Publicly available and reputable sources, such as the Android Malware Genome Project and Drebin dataset [34], were utilized to ensure diversity across a broad range of malware families. These datasets are widely recognized for their relevance in training machine learning models capable of generalizing across heterogeneous threat patterns. To enhance the balance and real-world applicability of the dataset, additional benign applications were sourced from trusted Android repositories, resulting in a well-distributed dataset that reflects practical deployment scenarios.

Let $D=\left\{d_{1},d_{2},\ldots ,d_{n}\right\}$ represent the total dataset, where each $d_{i}$ is an Android application classified as either benign or malicious. Let B⊂D denote the subset of benign applications, and M⊂D denote the subset of malicious applications. Thus, D=B∪M, where B∩M=∅. Ensuring a balanced distribution between B and M helps avoid class imbalance, which could bias the model towards predicting the majority class.

#### 3.1.2 Data preprocessing.

The preprocessing phase is a critical step to ensure data quality and consistency across all applications within the dataset. The preprocessing workflow includes the following stages: - In the preprocessing stage, each application $d_{i}\in D$ underwent a three-step transformation to ensure consistent and relevant data input for feature extraction and analysis. First, each APK was decompiled to access its internal code and resource structures, which are essential for capturing both static and dynamic features. This process is represented as $S_{i}=D\left(d_{i}\right)$, where $S_{i}$ denotes the set of decompiled features accessible for analysis and 𝒟 is the decompilation function. The decompilation step is critical for allowing the direct examination of permissions, API calls, and intent filters within the source code, all of which contribute to a static understanding of the behavior of the application. Following decompilation, a normalization process was applied to ensure a standardized format across all decompiled files, represented by $S_{i}^{\prime }=N\left(S_{i}\right)$. The normalization function N reformats diverse file types and code representations into a uniform structure, facilitating seamless and systematic analysis in the subsequent feature extraction phase. This step is crucial for minimizing inconsistencies across the dataset and enhancing compatibility with feature extraction algorithms, which require uniform input structures to process effectively.

### 3.2 Feature extraction and selection

To construct a robust Android malware detection framework, the feature extraction and selection processes were designed to capture both the static and dynamic attributes of applications.This dual-perspective approach enables the generation of a comprehensive behavioral profile for each sample, enhancing the model’s ability to discriminate between benign and malicious activities.

#### 3.2.1 Feature extraction.

Feature extraction was conducted to compile a complete set of relevant indicators, denoted by $=\left\{f_{1},f_{2},\ldots ,f_{k}\right\}$, for each application $d_{i}\in D$, where D is the full dataset. The features were categorized into static and dynamic subsets, reflecting the code-level properties and runtime behaviors, respectively.

##### A. Static features.

Static features, denoted $F_{\text{static\textasciitilde{}}}\subset F$, were extracted from the AndroidManifest.xml file and decompiled application source code. These include:

- Permissions (p): Requested access rights, such as camera, location, and network, which may suggest potential misuse or an elevated risk.
- API Calls (a): Specific function calls invoked by the application that may reveal operational intent or indicators of a compromise.
- Intent Filters (t): Declarations of inter-component communication mechanisms that can indicate the app’s interaction surface and potential misuse of Android’s messaging framework.

The static behavior of each application is formalized as shown in Eq (1):


$$S_{i}=\{p,a,t\},\text{\textasciitilde{}where\textasciitilde{}}p\in \text{\textasciitilde{}Permissions,\textasciitilde{}}a\in \text{\textasciitilde{}API Calls,\textasciitilde{}}t\in \text{\textasciitilde{}Intent Filters\textasciitilde{}}$$ (1)

The set of all static feature vectors was aggregated into a matrix $S=\left[S_{1},S_{2},\ldots ,S_{n}\right]$, where n is the number of applications analyzed.

**B. Dynamic features.** To characterize behavioral patterns during execution, dynamic features $F_{\text{dynamic\textasciitilde{}}}\subset F$ were collected in a controlled sandbox environment. These features encapsulate the real-time operational characteristics and consist of:

- System Calls (s): OS-level function invocations that provide insights into the internal activities of a program.
- Network Activity (n): Patterns of connectivity, including IP addresses, port usage, and transmission behavior, which may signal exfiltration or C2 activity.
- Inter-Process Communication (IPC) (c): Data exchange between application components or with external applications, often associated with privilege escalation or unauthorized data access [35].

The dynamic behavior of each application is modeled as shown in Eq (2):


$$D_{i}=\{s,n,c\},\text{\textasciitilde{}where\textasciitilde{}}s\in \text{\textasciitilde{}System Calls,\textasciitilde{}}n\in \text{\textasciitilde{}Network Activity,\textasciitilde{}}c\in IPC$$ (2)

The dynamic behavior across the dataset was compiled into a matrix $D=\left[D_{1},D_{2},\ldots ,D_{n}\right]$, capturing the sequential and behavioral diversity of application execution.The final feature representation used for model training was constructed by unifying both static and dynamic components:

#### 3.2.2 Feature selection.

Given the high dimensionality of the initial feature matrix F, feature selection techniques were applied to reduce redundancy, enhance computational efficiency, and preserve the discriminative power of the data.

A **Correlation-Based Filtering:** A correlation matrix $C\in {\mathbb{R}}^{k\times k}$ was computed, where each element $C_{ij}$ represents the Pearson correlation coefficient between feature pairs $f_{i}$ and $f_{j}$. Feature pairs exhibiting high correlation ($\left|C_{ij}\right|>\tau$, with τ as a predefined threshold) were identified and pruned to minimize multicollinearity. This process yielded a reduced feature subset $F_{\text{reduced\textasciitilde{}}}\subset F$, retaining only the most informative and statistically independent features. The reduction improved both model interpretability and training stability by eliminating redundant input signals.
B **Principal Component Analysis (PCA):** To further compress the feature space, PCA [36] was applied to the standardized feature matrix $X\in {\mathbb{R}}^{n\times k}$. The transformation is defined as (Eq.follows (3)):

Z=XW (3)

where $W\in {\mathbb{R}}^{k\times m}$ contains the eigenvectors of $X^{T}X$ associated with the top m eigenvalues, and $Z\in {\mathbb{R}}^{n\times m}$ represents the projection of the original features into an uncorrelated subspace. This transformation retains the maximum variance within the data while minimizing the information loss. The resulting matrix $F_{PCA}=Z$ provides a compact and expressive representation, thereby facilitating efficient and generalized training of the DBN-GRU model.

### 3.3 Feature vector generator

**Fig 2:**
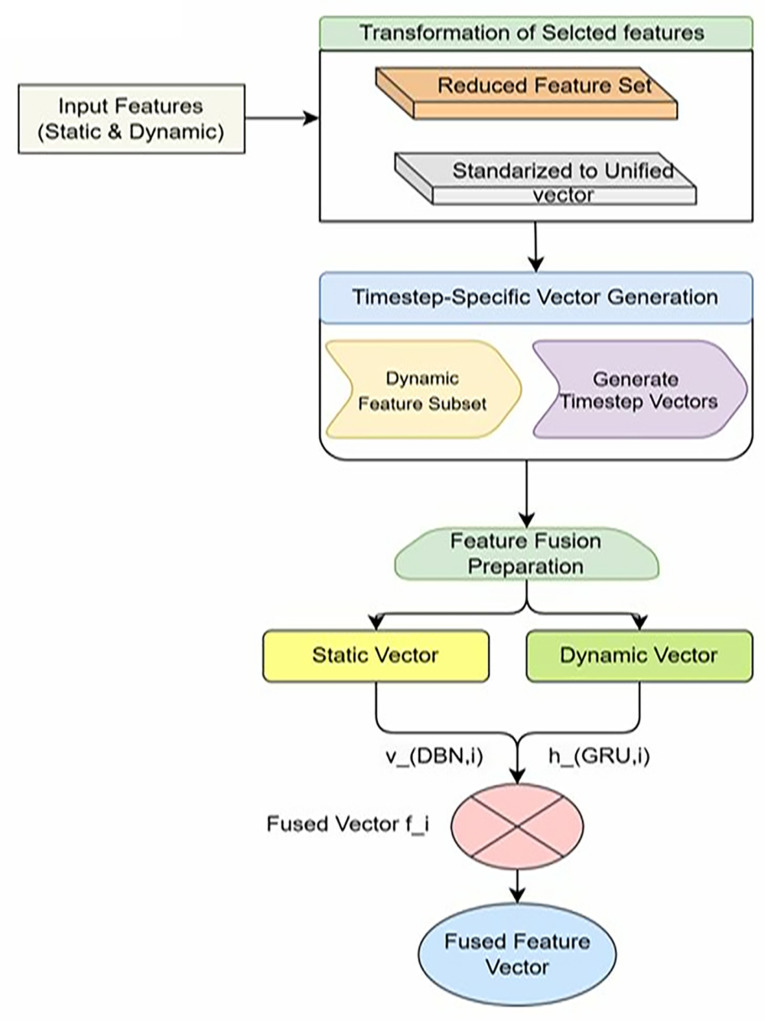
Flow of Feature Vector Generator.

The Feature Vector Generator serves as a critical intermediary in the Android malware detection pipeline, responsible for the systematic transformation and integration of both static and dynamic features into a unified vector representation. This vectorized input ensures that the DBN-GRU model receives a consistent and semantically enriched encoding of each application’s behavior, enabling robust and scalable classification.The operation of the Feature Vector Generator is structured into three primary stages, as illustrated in Fig 2: (i) transformation of selected features, (ii) generation of time-step specific vectors via GRU, and (iii) preparation for feature fusion.

#### Stage 1: Transformation of Selected Features.

 Following feature extraction and dimensionality reduction, the selected static and dynamic features were consolidated into a standardized vector format. Let $F_{\text{reduced\textasciitilde{}}}\subset F$ denote the refined feature set for application $d_{i}$, where $F_{\text{reduced\textasciitilde{}}}$ includes both static features (e.g., permissions, API calls, intent filters) and dynamic features (e.g., system calls, network activity). This set is formally defined as follows (Eq. (4)):


$$F_{\text{reduced\textasciitilde{}},i}=\left\{f_{i1},f_{i2},\ldots ,f_{im}\right\}$$ (4)

where m is the dimensionality of the reduced feature space and each $f_{ij}$ represents a selected feature for the $i^{\text{th\textasciitilde{}}}$ application.

The transformation function is then applied to generate a standardized vector $v_{i}$ as shown in Eq (5):


$$v_{i}=\text{\textasciitilde{}Transform\textasciitilde{}}\left(F_{\text{reduced\textasciitilde{}},i}\right)$$ (5)

Here, the Transform function standardizes and encodes each feature $f_{ij}$ into a format compatible with the DBN-GRU architecture. This transformation ensures consistency across all input vectors $v_{i}$, facilitating their seamless integration into the model’s fusion and classification layers.The resulting vector $v_{i}$ encapsulates both static indicators-such as code-level attributes-and dynamic behavioral signals derived from runtime analysis. This unified representation provides a comprehensive view of each application’s operational profile, supporting effective malware detection using the hybrid DBN-GRU framework.

#### Stage 2: Timestep-Specific vector generation for GRU.

A critical aspect of dynamic feature analysis is capturing temporal dependencies within sequential data. To address this, the Feature Vector Generator produces time-step-specific vectors for the GRU component, enabling the model to capture sequential patterns that may reveal malicious behaviors over time. Let $D_{\text{dynamic\textasciitilde{}},i}(t)$ denote the dynamic feature subset for application $d_{i}$ at timestep t. Each $D_{\text{dynamic,\textasciitilde{}}i}(t)$ includes temporal information, such as system calls and network activity, at specific points in the application’s runtime. The Feature Vector Generator processes this data to create a sequence of timestep-specific vectors $\left\{v_{i,1},v_{i,2},\ldots ,v_{i,T}\right\}$, where T is the total number of timesteps observed for application $d_{i}$.

Mathematically, each timestep vector $v_{i,t}$ is constructed as follows in Eq (6):


$$\begin{array}{c} {𝐯}_{i,t}=\text{ Transform}\left(D_{\text{dynamic\textasciitilde{}},i}(t)\right) \end{array}$$ (6)

Transform standardizations and structure the dynamic features of a time step t into a format that can be passed through the GRU.These time-step-specific vectors are then used by the GRU to model the temporal dependencies and learn the sequential correlations within the behavior of the application.Capturing behavioral patterns that evolve over time requires this process, which cannot be performed using static analysis alone.The Feature Vector Generator constructs these vectors such that the input to the GRU includes both immediate and historical behaviors, making it possible for the model to distinguish between benign and malicious sequences.

#### Stage 3: Feature fusion preparation.

The feature fusion data are the final features of the Feature Vector generator.A fused representation that merges static and dynamic features is required for the hybrid DBN-GRU model to fully utilize an application’s behavioral profile.This is achieved by the Feature Vector Generator, which combines the static feature representation (processed by the DBN) and the sequence of time step-specific dynamic vectors (processed with the GRU).Let $v_{DBN,i}$ denote the high-dimensional vector produced by the DBN for the static features of application $d_{i}$, and $h_{GRU,i}$ denote the final hidden state from the GRU, which encapsulates temporal dependencies from the dynamic features. The fused feature vector $f_{i}$ for application $d_{i}$ is then constructed asshown in Eq (7):


$$\begin{array}{c} {𝐟}_{i}={𝐯}_{DBN,i}⊕{𝐡}_{GRU,i} \end{array}$$ (7)

Where from the Eq (6) ⊕ denotes the concatenation operation. These fusionscombine both static code-based features and dynamic behavior-based features into a single comprehensive vector, $f_{i}$, which encapsulates the multidimensional characteristics of the application. The fused vector $f_{i}$ is then fed into the classification layer for final categorization. This layer, using a softmax activation function, computes the probability P (class $=c|f_{i}$) for each class (benign or malicious) as shown in Eq (8):


$$\begin{array}{c} P\left(\text{\textasciitilde{}class\textasciitilde{}}=c|{𝐟}_{i}\right)=\frac{exp\left(W_{c}^{⊤}{𝐟}_{i}+b_{c}\right)}{\sum_{j}\;\;exp\left(W_{j}^{⊤}{𝐟}_{i}+b_{j}\right)} \end{array}$$ (8)

From the above Eq (8) where $W_{c}$ and $b_{c}$ are the weights and biases associated with class c. The class with the highest probability is selected as the predicted label for application $d_{i}$.

The Feature Vector Generator plays an important role in preparing unified and structured inputs for the DBN-GRU model. The Feature Vector Generator ensures that the model takes full and compatible input by transforming different features into standardized vectors, generating step-specific time representations for the GRU, and preparing the data for feature fusion.The DBN-GRU model performs this process well, allowing it to use both static and dynamic features effectively and achieve a robust Android malware detection model that can detect fine-grained malicious behaviors in various applications.

### 3.4 Proposed DBN-GRU model architecture

**Fig 3:**
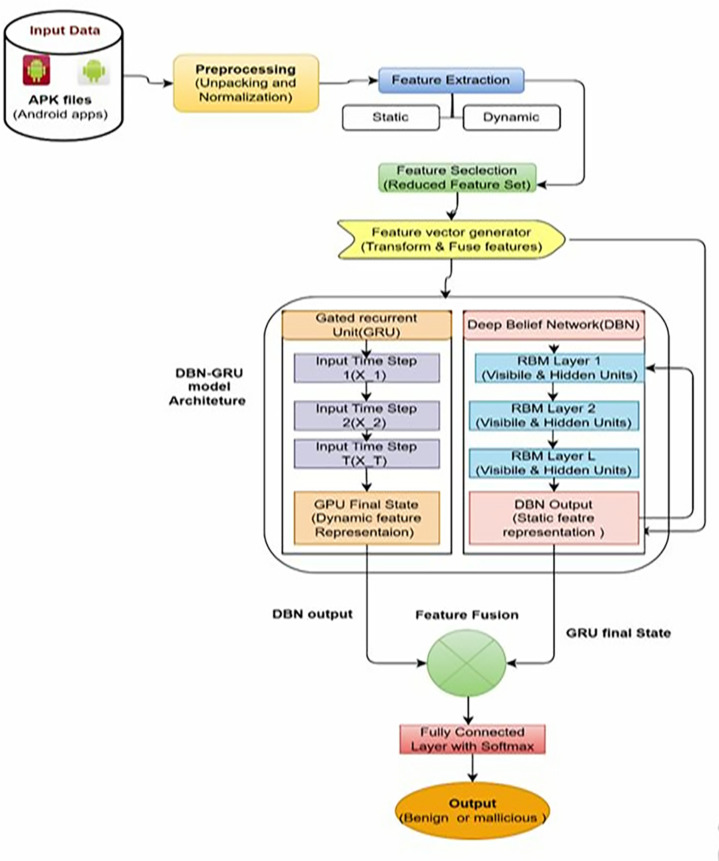
Overall Flow diagram of the ProposedDBN-GRU model. Finally, we create that the hybrid DBN-GRU model architecture simultaneously extracts unsupervised features for Android applications with sequential dependency model using GRU to enhance the analysis of Android applications in a comprehensive manner. We demonstrate this approach can identify sophisticated malware patterns and significantly improve detection accuracy in dynamic Android environments.

A hybrid DBN–GRU architecture that combines the power of static and dynamic feature analyses to detect Android malware is proposed.The architecture is a combined architecture that takes advantage of the ability of DBNs [37] to extract hierarchical features and GRUs to model temporal sequences to obtain a robust system to capture intricate malware behaviors over multiple dimensions, as shown in Fig 3.

#### 3.4.1 Deep Belief Network (DBN).

The DBN provides the first layer of architecture to perform unsupervised hierarchical feature extraction. Thus, the system consists of several layers of Restricted Boltzmann Machines (RBMs) [38], which are probabilistic neural networks that learn the representations of input data through unsupervised training [39]. This allows each of its layers to progressively learn increasingly abstract features, which in turn allows the model to discover complex patterns for distinguishing benign from malignant applications.

In the layer configuration phase, The DBN architecture is composed of L stacked Restricted Boltzmann Machine (RBM) layers, where each layer ${RBM}_{l}$ for l=1,2,…,L consists of a visible layer v and a corresponding hidden layer h. These layers are arranged hierarchically, such that the output of each RBM serves as the input to the subsequent layer. This configuration facilitates the progressive abstraction of feature representations, enabling the DBN to learn increasingly complex and high-level patterns from the input data.The probabilistic behavior of each RBM layer is characterized by the joint distribution between the visible and hidden units, as defined in Equation (9).


$$\begin{array}{c} P(𝐯,𝐡)=\frac{1}{Z}exp(-E(𝐯,𝐡)) \end{array}$$ (9)

where Z is the partition function and E(v,h) represents the energy function, defined as in Eq (10):


$$\begin{array}{c} E(𝐯,𝐡)=-{𝐯}^{⊤}Wh-{𝐛}^{⊤}𝐯-{𝐜}^{⊤}𝐡 \end{array}$$ (10)

Here in Eq (9), W is the weight matrix between visible and hidden units, and b and c are the biases for the visible and hidden layers, respectively.

The pre-training phase of the DBN involves unsupervised learning within each Restricted Boltzmann Machine (RBM) layer to extract meaningful feature representations. This process is conducted sequentially, beginning with the lowest layer and progressing upward through the network. Contrastive divergence is employed to minimize the divergence between the observed and reconstructed data distributions, enabling each RBM to effectively learn the underlying structure of the input space. By stacking RBMs in this hierarchical manner, the DBN captures intricate patterns and produces high-dimensional static feature representations that encapsulate complex behaviors associated with both benign and malicious applications. These enriched static features are subsequently complemented by dynamic behavioral features, which are processed by the GRU component to capture temporal dependencies within the application activity sequences.

#### 3.4.2 Gated Recurrent Unit (GRU).

To handle sequential dependencies within the dynamic feature set, which is necessary to map temporal correlations that might suggest malicious behavior over time, the GRU component is incorporated. The GRU is a recurrent neural network specifically designed to handle long-term dependencies and overcome the vanishing gradient problem that affects traditional recurrent neural networks.

A **Sequence Modeling:**The GRU processes dynamic features as temporal sequences, with each time step representing a distinct point in the application’s behavior. Let $x_{t}$ represent the input feature vector at timestep t, and $h_{t}$ the hidden state. The GRU maintains and updates its hidden state over time, enabling it to capture dependencies between sequential events.The update equations for the GRU are as follows (Eq (11)):

$$\begin{array}{c} \begin{array}{c} {𝐳}_{t}=\sigma \left({𝐖}_{z}{𝐱}_{t}+{𝐔}_{z}{𝐡}_{t-1}+{𝐛}_{z}\right)\\ {𝐫}_{t}=\sigma \left({𝐖}_{r}{𝐱}_{t}+{𝐔}_{r}{𝐡}_{t-1}+{𝐛}_{r}\right)\\ {𝐡}_{t}=\left(1-{𝐳}_{t}\right)⊙{𝐡}_{t-1}+{𝐳}_{t}⊙tanh\left({𝐖}_{h}{𝐱}_{t}+{𝐔}_{h}\left({𝐫}_{t}⊙{𝐡}_{t-1}\right)+{𝐛}_{h}\right) \end{array} \end{array}$$ (11)

From the above Eq (8) $z_{t}$ and $r_{t}$ are the update and reset gates, respectively, controlling how information flows through the network, and σ denotes the sigmoid function.

B **Integration with DBN**As input, the DBN sends feature representations to the GRU, which can then fuse static hierarchical features with dynamic temporal sequences.With this integration, both types of features can be used simultaneously to capture complex behavioral patterns that span both static code properties and temporal dynamics.Temporal dependencies through the dynamic features of an application’s deep dynamic features are encapsulated in the final hidden state of the GRU, distinguishing benign from malicious sequences.

#### 3.4.3 Hybrid DBN-GRU Model.

The DBN and GRU components are integrated into a unified hybrid architecture designed to leverage both static and dynamic feature representations, thereby enhancing the robustness and effectiveness of the Android malware detection.

A **Feature Fusion:**The merging of the output feature representations from the static features (DBN) and dynamic features (GRU) results in a comprehensive feature vector that captures the behavior of the application in different dimensions.The fused representation of the application in the model provides a view of the application at the code and runtime levels.
B **Classification Layer:**The final feature vector f is passed through a fully connected classification layer with a softmax activation function, which outputs a probability distribution over the classes (benign or malicious). Let $W_{fc}$ and $b_{fc}$ denote the weights and biases of the fully connected layer. The probability P( class ∣f) for each class is given in Eq (12).

$$\begin{array}{c} P(\text{\textasciitilde{}class\textasciitilde{}}=c|𝐟)=\frac{exp\left({𝐖}_{fc,c}^{⊤}𝐟+{𝐛}_{fc,c}\right)}{\sum_{j}\;\;exp\left({𝐖}_{fc,j}^{⊤}𝐟+{𝐛}_{fc,j}\right)} \end{array}$$ (12)

Where in Eq (12) c∈{ benign, malicious }. The class with the highest probability is chosen as the model’s prediction, classifying the application accordingly.

#### 3.4.4 Algorithm: DBN-GRU based android malware detection model.

- **Input:**$D=\left\{d_{1},d_{2},\ldots ,d_{n}\right\}$: A dataset of Android APK files, labeled as benign or malicious.
- **Output:**Classification Result: A binary label (benign or malicious) for each application $d_{i}$.

##### Steps:

###### Step 1: Data collection and preprocessing

- Collect APK files and label each file as benign or malicious, creating subsets B⊂D (benign) and M⊂D (malicious) such that D=B∪M and B∩M=∅.
- For each APK $d_{i}$, decompile to obtain the code structure $S_{i}=D\left(d_{i}\right)$.
- Normalize $S_{i}$ using a function $S_{i}^{\prime }=N\left(S_{i}\right)$ to ensure consistency across samples.
- Result: Preprocessed dataset $D^{\prime }=\left\{S_{1}^{\prime },S_{2}^{\prime },\ldots ,S_{n}^{\prime }\right\}$.

###### Step 2: Feature Extraction

- *Static Features*: For each application $S_{i^{\prime }}^{\prime }$ extract static features $S_{i}=\{p,a,t\}$, where: p: Permissions, a: API calls, t: Intent filters.
- *Dynamic Features:* Collect dynamic features $D_{i}=\{s,n,c\}$, where: s: System calls, n: Network activity, c: Inter-process communications (IPC).
- Result: Combined feature set F=S∪D.

###### Step 3: Feature Selection

- Correlation Analysis: Calculate the correlation matrix C for all features in F and remove features with high correlations.
- Principal Component Analysis (PCA): Transform the remaining features into a set of orthogonal components: Z=XW, where W contains eigenvectors of $X^{T}X$.
- Select the top m components to create the reduced feature set $F_{\text{reduced\textasciitilde{}}}=\left\{f_{i1},f_{i2},\ldots ,f_{im}\right\}$.
- Result: $F_{\text{reduced,\textasciitilde{}}}$ a reduced feature set with minimal redundancy.

###### Step 4: Feature Vector Generation

- Transform Static Features: Standardize static features into a unified vector $v_{\text{static\textasciitilde{}},i}$ for each application.
- Generate Timestep-Specific Vectors: For each dynamic feature subset $D_{\text{dynamic,\textasciitilde{}}i}(t)$ at timestep t, create timestep-specific vectors $v_{\left(i,t\right)}=Transform\left(D_{\text{dynamic\textasciitilde{}},i}(t)\right)$.
- Feature Fusion Preparation: Combine the static and dynamic representations to create a comprehensive feature vector $v_{i}$ for each application:

$$v_{i}=v_{\text{static\textasciitilde{}},i}⊕\left(v_{\left(i,1\right)},v_{\left(i,2\right)},\ldots ,v_{\left(i,T\right)}\right)$$ (13)

- Result: Unified feature vector $v_{i}$ for each application.

###### Step 5: DBN Training

A Deep Belief Network (DBN) is configured with L stacked layers of Restricted Boltzmann Machines (RBMs), each responsible for learning hierarchical feature abstractions from the input data. For each layer l∈{1,2,…,L}, training is conducted using the contrastive divergence algorithm to minimize reconstruction error and capture latent representations effectively.

The joint probability distribution between the visible and hidden units in an RBM layer is defined as follows (Eq. (14)):


$$\begin{array}{c} P(v,h)=\frac{1}{Z}\text{exp}(-E(v,h)) \end{array}$$ (14)

where the energy function E(v,h) is given by:$E(v,h)=-v^{T}Wh-b^{T}v-c^{T}h$, Here, W denotes the weight matrix, while b and c represent the bias vectors for the visible and hidden layers, respectively. This formulation enables each RBM to learn meaningful feature dependencies in an unsupervised way.Upon completion of layer-wise training, the DBN outputs a high-dimensional static feature representation for each input instance, denoted as $v_{DBN,i}$. This representation encapsulates the abstract, non-linear patterns essential for distinguishing between benign and malicious applications.

###### Step 6: GRU Processing

- The GRU parameters are initialized to model the sequential dependencies of the dynamic features.
- For each timestep t, update the GRU hidden state $h_{t}$ using Eq (11).
- Capture the final hidden state $h_{GRU,i}=h_{T}$.
- Result: Temporal dynamic feature representation $h_{GRU,i}$ from the GRU.

###### Step 7: Feature fusion and classification

- Concatenate the DBN output $v_{DBN,i}$ and the GRU final state $h_{GRU,i}$ to create a fused feature vector $f_{i}$:$f_{i}=v_{DBN,i}⊕h_{GRU,i}$
- Pass $f_{i}$ through a fully connected layer with softmax activation to predict the class label as Show in Eq (12).
- Choose the class c with the highest probability as the final prediction.

###### End of Algorithm

**Algorithm 1** presents the step-by-step methodology of the proposed DBN-GRU-based Android malware detection model. An algorithm is presented that starts with data collection and preprocessing, where Android APK files are labeled and standardized (for consistency). After feature extraction, the features are placed into static and dynamic categories to comprehensively capture the application behavior.Dimensionality was reduced by applying feature selection using correlation analysis and Principal Component Analysis (PCA), keeping only the most relevant features.Static features are standardized, dynamic features are represented at each time step, and a unified feature vector is generated.The DBN is then trained to learn hierarchical static feature representations and GRU dynamic features to examine temporal dependencies.Finally, the final feature vector was input into a classification layer with a SoftMax activation to predict whether the input was benign or malicious for each application.They then fused the DBN and GRU outputs.

### 3.5 Training and validation

A supervised learning process was applied to the training and validation stages of the hybrid DBN GRU model, so that the model can classify applications as benign/malicious and generalize.

A **Dataset Splitting:** The dataset was stratified into three subsets–training, validation, and test sets–with a 70:15:15 split.This division was meant to allow a rich evaluation in which the subsets had roughly equal representations of benign and malicious samples.The model parameters were optimized using the training set, and the validation set was used to perform hyperparameter tuning in an unbiased manner.The final model was used solely to test the performance of the model on the test set, which was held back from training and tuning.
B **Optimization:** Model training was performed using the Adam optimizer, which was selected for its adaptive learning rate capabilities, which improved the convergence speed and stability. The learning rate was set to α=0.001, with the optimizer minimizing the categorical cross-entropy loss function as shown in Eq (15):

$$\begin{array}{c} ℒ=-\sum_{i=1}^{C}\;y_{i}\text{log}\left({\hat{y}}_{i}\right) \end{array}$$ (15)

where $y_{i}$ is the true label and ${\hat{y}}_{i}$ is the predicted probability for each class i in the set of classes C. From the above equation (15).This loss function penalizes incorrect predictions and guides the model toward a more accurate classification.

C. **Regularization:** In the DBN-GRU architecture, dropout layers are introduced to address overfitting.Averaging over multiple channels removes the need for individual neurons to perform extra work, but also means that the model cannot get too lazy: the neurons in these layers are randomly deactivated with a given probability p during training.Such a regularization technique helps the model to have generalized feature representations, which can be helpful for classification when malware patterns are complex and varied.Their dropout probability was tuned to achieve a trade-off between the model complexity and generalization.
D. **Validation:** The model performance was validated iteratively using the validation set throughout the training.For example, the validation loss and classification accuracy were monitored to adjust the hyperparameters, such as the number of layers, dropout rate, and learning rate.The model parameters were tuned during the validation process to optimize the generalization performance, free from overfitting to the training data, and to improve the model accuracy on unseen samples.

This structured training and validation approach enabled the hybrid DBN-GRU model to achieve a high classification accuracy, providing a balanced and resilient model for Android malware detection.

### 3.6 Evaluation metrics

The hybrid DBN-GRU model’s performance in classifying Android applications as benign or malicious was assessed using a comprehensive set of evaluation metrics to provide insights into its precision, sensitivity, and overall discriminative ability.Accuracy reflects the proportion of correctly classified instances to the total evaluated, as shown in Eq (16):


$$\text{Accuracy\textasciitilde{}}=\frac{TP+TN}{TP+TN+FP+FN}$$ (16)

While informative, accuracy alone may not suffice for imbalanced datasets in which false positives and false negatives vary significantly.

Precision measures the model’s ability to correctly identify true malicious instances, as shown in Eq (17):


$$\text{Precision\textasciitilde{}}=\frac{TP}{TP+FP}$$ (17)

High precision indicates a low false-positive rate, reducing incorrect malware detection.

Recall (sensitivity) quantifies the model’s capability to capture actual malicious instances, as shown in Eq (18):


$$\text{Recall\textasciitilde{}}=\frac{TP}{TP+FN}$$ (18)

A high recall rate is crucial for effective malware detection in security applications.

F1-Score, the harmonic mean of precision and recall, balances both metrics, as Shown in Eq (19):


$$\text{F1-Score\textasciitilde{}}=2\times \frac{\text{\textasciitilde{}Precision\textasciitilde{}}\times \text{\textasciitilde{}Recall\textasciitilde{}}}{\text{\textasciitilde{}Precision\textasciitilde{}}+\text{\textasciitilde{}Recall\textasciitilde{}}}$$ (19)

This metric is valuable for addressing imbalanced datasets.

The Area Under the Receiver Operating Characteristic Curve (AUC-ROC) evaluates the model’s ability to distinguish between benign and malicious classes, as shown In Eq (20):


$$AUC=\int_{0}^{1}\;ROC(p)dp$$ (20)

A high AUC-ROC score indicates robust model discrimination, irrespective of the threshold settings.

#### Performance analysis metrics.

**The Feature Extraction Time**(Table 4) evaluates the average time required to extract features, indicating computational efficiency from the following Eq (21):

**Table 2:** Dataset composition.

Application Type	Number of Samples
Benign	123,453
Malicious	5,560
**Total**	129,013

**Table 3:** Performance comparison of proposed and baseline approaches based on metrics.

Model	Accuracy (%)	Precision (%)	Recall (%)	F1-score (%)	AUC
DBN-GRU(Proposed)	98.7	98.5	98.9	98.7	0.99
NMLA-AMDCEF [12]	94.4	93.8	94.1	93.9	0.95
MalVulDroid [13]	95.2	94.7	95.0	94.8	0.96
LinRegDroid [14]	93.6	93.1	93.4	93.2	0.94

**Table 4:** Preprocessing accuracy levels.

Records Considered	DBN-GRU Model (%)	NMLA-AMDCEF (%) [12]	MalVulDroid (%) [13]	LinRegDroid (%) [14]
10,000	97.4	93.4	94.3	92.6
20,000	97.6	93.7	94.5	92.9
30,000	97.8	93.9	94.7	93.0
40,000	98.0	94.1	94.9	93.2
50,000	98.1	94.3	95.0	93.4
60,000	98.2	94.4	95.2	93.6


$$\text{Feature Extraction Time\textasciitilde{}}=\frac{\text{\textasciitilde{}Total time for feature extraction\textasciitilde{}}}{\text{\textasciitilde{}Number of records\textasciitilde{}}}$$ (21)

**Feature Selection Accuracy** (Table 5) measures the accuracy of identifying relevant features, as shown in Eq (22):

**Table 5:** Feature extraction time levels (in seconds).

Records Considered	DBN-GRU Model	NMLA-AMDCEF (%) [12]	MalVulDroid (%) [13]	LinRegDroid (%) [14]
10,000	16.8	21.6	22.4	21.1
20,000	17.1	21.8	22.6	21.2
30,000	17.3	22.0	22.8	21.3
40,000	17.6	22.1	23.0	21.5
50,000	17.9	22.4	23.1	21.6
60,000	18.0	22.6	23.2	21.8


$$\text{Feature Selection Accuracy\textasciitilde{}}=\frac{\text{\textasciitilde{}Number of correctly selected relevant features\textasciitilde{}}}{\text{\textasciitilde{}Total number of features\textasciitilde{}}}\times 100\;\%$$ (22)

**Feature Dimensionality Reduction Accuracy** (Table 6) assesses the preservation of essential information after reducing the feature count from the following equation (23):

**Table 6:** Feature selection accuracy levels.

Records Considered	DBN-GRU Model (%)	NMLA-AMDCEF (%) [12]	MalVulDroid (%) [13]	LinRegDroid (%) [14]
10,000	97.9	91.9	93.1	92.9
20,000	98.0	92.0	93.2	93.1
30,000	98.1	92.1	93.4	93.5
40,000	98.3	92.4	93.5	93.8
50,000	98.5	92.6	93.7	94.0
60,000	98.7	92.7	93.9	94.2


$$\text{Dimensionality Reduction Accuracy\textasciitilde{}}=\frac{\text{\textasciitilde{}Information retained post-reduction\textasciitilde{}}}{\text{\textasciitilde{}Original total information\textasciitilde{}}}\times 100\;\%$$ (23)

**Feature Processing Accuracy (**Table 7) evaluates the model ‘s accuracy in maintaining feature integrity, as shown in Eq (24).

**Table 7:** Feature dimensionality reduction accuracy levels.

Records Considered	DBN-GRU Model (%)	NMLA-AMDCEF (%) [12]	MalVulDroid (%) [13]	LinRegDroid (%) [14]
10,000	97.6	93.1	92.5	92.7
20,000	97.9	93.2	92.7	92.9
30,000	98.1	93.5	92.9	93.1
40,000	98.3	93.7	93.1	93.4
50,000	98.5	93.9	93.4	93.6
60,000	98.6	94.0	93.6	93.8


$$\text{Feature Processing Accuracy\textasciitilde{}}=\frac{\text{\textasciitilde{}Number of correctly processed feature sets\textasciitilde{}}}{\text{\textasciitilde{}Total feature sets\textasciitilde{}}}\times 100\;\%$$ (24)

**Malware Detection Time** (Table 8) measures the time taken to classify records, indicating real-time performanceas shown in Eq (25):

**Table 8:** DBN-GRU feature processing accuracy levels.

Records Considered	DBN-GRU Model (%)	NMLA-AMDCEF (%) [12]	MalVulDroid (%) [13]	LinRegDroid (%) [14]
10,000	97.9	92.3	91.7	93.7
20,000	98.1	92.5	91.8	93.9
30,000	98.3	92.7	92.0	94.1
40,000	98.5	92.9	92.3	94.3
50,000	98.7	93.1	92.5	94.4
60,000	98.8	93.3	92.7	94.6


$$\text{Detection Time\textasciitilde{}}=\frac{\text{\textasciitilde{}Total time taken for detection\textasciitilde{}}}{\text{\textasciitilde{}Number of records\textasciitilde{}}}$$ (25)

**The Malicious Signal Detection Accuracy** (Table 9) quantifies the precision of the model in identifying true malicious signals, as Shown in Eq (26).

**Table 9:** Malware detection time levels (in seconds).

Records Considered	DBN-GRU Model (proposed)	NMLA-AMDCEF (%) [12]	MalVulDroid (%) [13]	LinRegDroid (%) [14]
10,000	16.2	22.9	24.3	22.0
20,000	16.3	23.0	24.5	22.1
30,000	16.5	23.1	24.7	22.3
40,000	16.7	23.2	24.8	22.5
50,000	16.8	23.3	25.0	22.7
60,000	17.0	23.6	25.2	22.8


$$\text{Malicious Detection Accuracy\textasciitilde{}}=\frac{\text{\textasciitilde{}Number of true positives and true negatives for malicious signals\textasciitilde{}}}{\text{\textasciitilde{}Total number of records evaluated\textasciitilde{}}}\times 100\;\%$$ (26)

These metrics collectively provide a robust evaluation model, confirming the applicability and performance of the model for real-world Android malware-detection.

## 4. System specifications and model implementation

Using an Intel Core i7 processor, 16GB RAM, and an NVIDIA GTX 1080 GPU system, the Android malware detection model was implemented using the proposed DBN GRU-based model. Python was chosen to develop the model using the popular Python libraries TensorFlow and Keras, which were used to construct and train the GRU and DBN architectures, respectively.Custom scripts for data preprocessing, feature extraction, and selection were automated, and dynamic analysis in a controlled virtual environment was employed to capture runtime behaviors.The static features were learned layer by layer using unsupervised learning to form hierarchical representations, whereas the dynamic features were processed sequentially by the GRU to learn their temporal dependencies.To guarantee robustness and generalizability across diverse malware families, we fine-tuned the model and evaluated it using stratified cross-validation.

### 4.1 Model hyperparameters and training configuration

The hyperparameters of the DBN and GRU components were meticulously configured to optimize the overall model performance.For the DBN, a three-layer architecture was employed, comprising successive Restricted Boltzmann Machine (RBM) layers with 128, 64, and 32 hidden units.This hierarchical structure facilitates progressive feature abstraction and contributes to the model’s ability to capture complex patterns in input data.Each RBM was pre-trained using the (CD-1) step and learned at an initial rate of 0.01.The number of units in the hidden layer of the GRU used for training was 64, and a dropout rate of 0.2 was used to avoid overfitting.In this case, the GRU was trained using a learning rate of 0.001 with an Adam optimizer.Additional training settings were also added, with a batch size of 32 and an early stopping criterion that did not allow overfitting and used a patience of 10 epochs. We chose these hyperparameters through a combination of grid search and empirical testing to provide a balance between the computational efficiency and detection accuracy.The dataset used in this study was the Drebin Dataset, an Android application dataset compiled by Technosphere Universität Braunschweig.We present this dataset, which consists of 5,560 malware samples from 179 distinct families, collected between August 2010 and October 2012.For Android malware detection, 123,453 benign applications were also included, providing a balanced dataset for the training and evaluation of the proposed DBN-GRU model, as shown in Table 2.This dataset is publicly available and has been widely used to benchmark malware detection methodologies in academic research.

The proposed detection model was developed and assessed on a robust and representative basis using this dataset, given its extensive coverage of malware families and numerous benign samples.

## 5. Results and discussion

### 5.1 Overview of classification performance

As Android malware becomes increasingly sophisticated, often obfuscating behavior or exploiting zero-day vulnerabilities, traditional detection models face growing limitations.Techniques based on static rules or shallow machine learning often struggle to keep pace, particularly when they are required to identify subtle, evolving attack patterns.

The proposed DBN-GRU model addresses these shortcomings by combining static code-level insights with dynamic behavioral patterns captured during execution. Through this hybrid architecture, the model builds a deeper understanding of application behavior by integrating the abstraction capabilities of DBNs with the sequence modeling ability of GRUs.

Rather than relying on fixed feature sets, the model adapts to varying forms of malware by learning from both structural indicators (e.g., permissions and API calls) and runtime signals (e.g., system calls and network activity).This layered approach improves the ability to detect previously unseen threats and better differentiates malicious behavior from benign anomalies.

Compared to established models such as NMLA-AMDCEF, MalVulDroid, and LinRegDroid, the DBN-GRU architecture demonstrated higher classification accuracy and greater efficiency in feature processing. These results reflect the enhanced generalization capacity of the model and its potential to support real-time detection in modern mobile environments.

### 5.2 Performance metrics and comparative analysis

To rigorously evaluate the proposed DBN-GRU model for Android malware detection, a comprehensive suite of performance metrics was employed: accuracy, precision, recall, F1-score, and area under the curve (AUC).These indicators offer a multidimensional understanding of the model’s effectiveness in classifying benign and malicious applications.

The DBN-GRU model achieved superior classification effectiveness, as evidenced by a comprehensive set of performance metrics.An accuracy of 98.7% reflects its capability to reliably distinguish between benign and malicious applications.The precision score of 98.5% demonstrates the model’s efficacy in minimizing false-positive detections, which is critical for reducing unnecessary alerts in operational environments.A recall of 98.9% highlights its strong sensitivity to actual threats, ensuring minimal oversight of malicious activity.The F1-score, calculated at 98.7%, indicates an optimal balance between precision and recall, confirming the robustness of the model under diverse threat conditions. Moreover, an AUC value of 0.99 underscores its high discriminative capacity across all decision thresholds, affirming the model’s effectiveness in maintaining performance consistency under varying classification criteria.Compared with traditional models, such as NMLA-AMDCEF, MalVulDroid, and LinRegDroid, the DBN-GRU consistently surpassed them in all performance metrics, as shown in Table 3.

In the comparative analysis, the DBN-GRU model consistently exceeded the performance of traditional benchmarks, offering notable gains in both accuracy and discriminative power.While existing approaches such as NMLA-AMDCEF, MalVulDroid, and LinRegDroid demonstrated moderate effectiveness, their limitations in handling complex malware patterns became evident through lower precision and AUC values.The substantial margin of improvement of the DBN–GRU across all metrics highlights its architectural advantage in capturing nuanced static and dynamic behavioral traits, thereby offering a more resilient and generalizable framework for contemporary Android malware detection.

**Fig 4:**
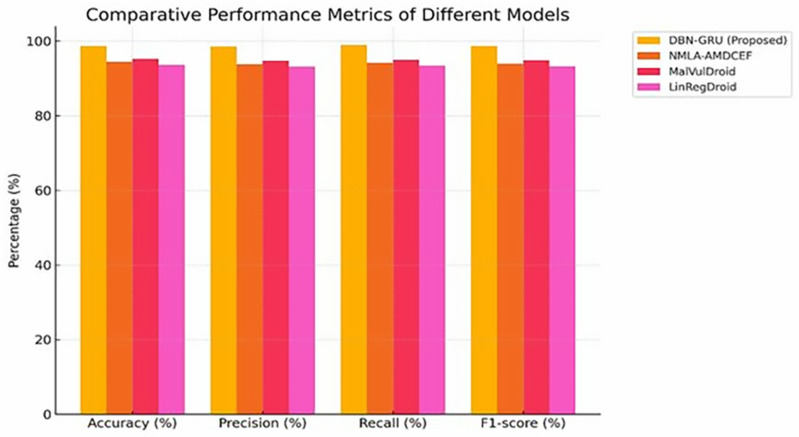
Comparative Performance Metrics Analysis of DBN-GRU and Traditional models.

Fig 4 presents a consolidated comparison of the core performance metrics across all evaluated models, highlighting the consistent dominance of DBN-GRU in terms of classification accuracy, precision, recall, and F1-score.The visual trend underscores the proposed model’s capacity to maintain high detection reliability while minimizing the false classifications.Notably, this comparative gap reinforces the architectural advantages of the DBN-GRU framework over conventional approaches, particularly in scenarios requiring robust generalization across diverse malware behaviors.

**Fig 5:**
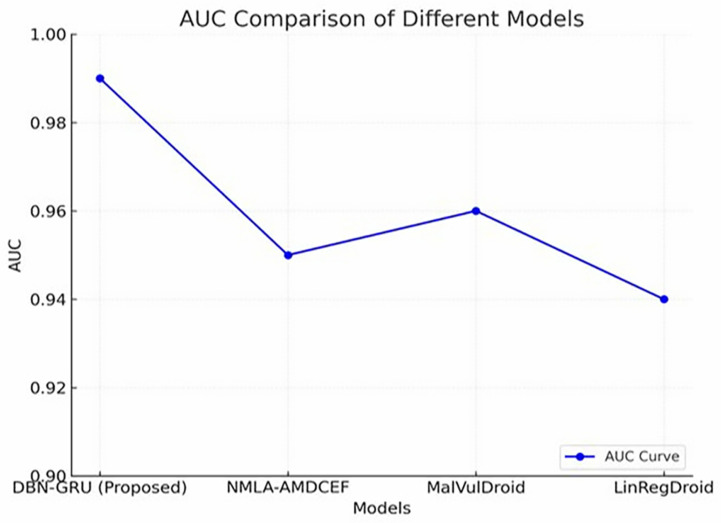
AUC Comparison Curve for Different models.

Fig 5 illustrates the AUC comparison between the proposed DBN-GRU model and the established baselines.The DBN-GRU model attained the highest AUC score of 0.99, indicating its exceptional capability in distinguishing between benign and malicious applications across varying decision thresholds.In contrast, the traditional models (MalVulDroid, NMLA-AMDCEF, and LinRegDroid) exhibited comparatively lower separability, as reflected by AUC scores of 0.96, 0.95, and 0.94, respectively.This significant margin further substantiates the effectiveness of the DBN-GRU model in handling complex classification boundaries, affirming its suitability for precise and reliable malware detection in dynamic mobile environments.

#### 5.2.1 Preprocessing accuracy.

Preprocessing accuracy plays a critical role in determining the reliability of subsequent machine learning tasks because it directly influences the integrity of the feature representation.

As reported in Table 4, the DBN-GRU model demonstrated a consistent upward trend in preprocessing accuracy across increasing dataset sizes, ranging from 97.4% at 10,000 records to 98.2% at 60,000 records.In contrast, baseline models, including NMLA-AMDCEF, MalVulDroid, and LinRegDroid, exhibited lower and relatively flatter performance curves, with maximum accuracy values plateauing between 93.6% and 95.2%.

**Fig 6:**
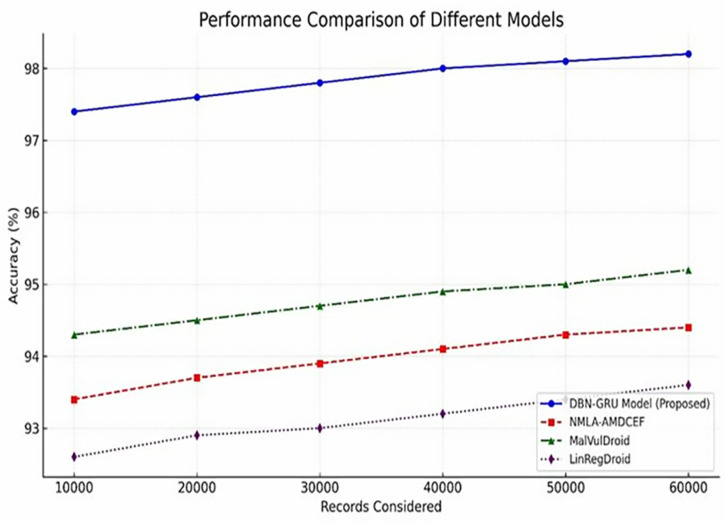
Preprocessing Accuracy Levels.

This disparity, further visualized in Fig 6, reinforces the enhanced capability of the DBN–GRU model to process diverse and large-scale datasets with minimal degradation in data fidelity.Such robustness during preprocessing ensures that the model maintains a high input quality, thereby strengthening the overall detection reliability in operational environments.

#### 5.2.2 Feature extraction time.

Timely feature extraction is essential for operational malware detection systems, particularly those requiring near-real-time responsiveness.

As detailed in Table 5, the DBN-GRU model consistently achieved reduced feature extraction latency across all dataset scales. Specifically, it processed 60,000 records in just 18.0 s, outperforming NMLA-AMDCEF (22.6 s), MalVulDroid (23.2 s), and LinRegDroid (21.8 s).

**Fig 7:**
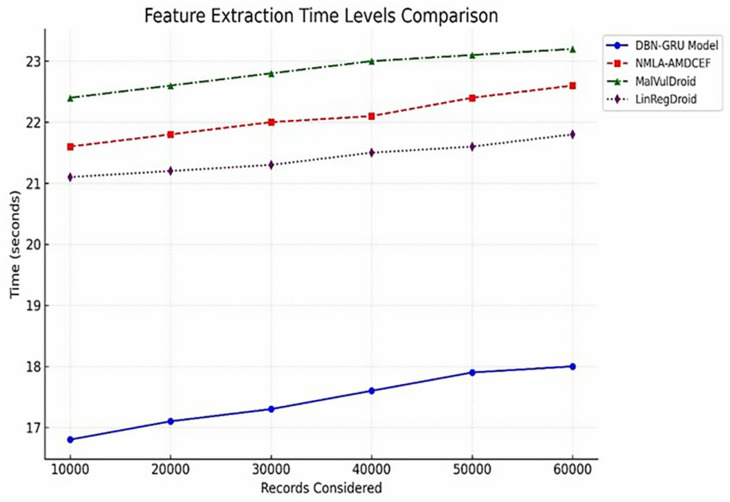
Feature Extraction Time Levels.

This performance advantage, further illustrated in Fig 7, underscores the architectural efficiency of the DBN-GRU framework in streamlining the extraction of both static and dynamic features. The model’s lower computational burden during this stage not only accelerates the detection pipeline but also makes it highly suitable for deployment in environments in which responsiveness and throughput are critical.

#### 5.2.3 Feature selection accuracy.

Feature selection plays a pivotal role in reducing dimensional noise while preserving discriminative power, thereby enhancing the overall model performance. As indicated in Table 6

The DBN-GRU model consistently demonstrated superior accuracy in identifying relevant features across all dataset sizes, achieving 98.7% accuracy with 60,000 records.In contrast, traditional models, NMLA-AMDCEF, MalVulDroid, and LinRegDroid, exhibited comparatively lower performance, with their feature selection accuracy peaking below 94.2%.

**Fig 8:**
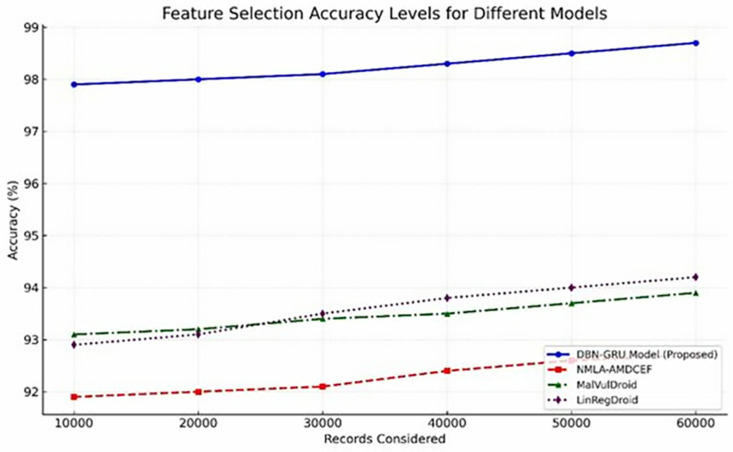
Feature Selection Accuracy Levels.

This consistent margin, illustrated in Fig 8, highlights the effectiveness of the DBN-GRU framework in isolating the most informative attributes critical to malware detection.By prioritizing features with high predictive values, the model optimizes its detection capability while simultaneously reducing computational redundancy, further strengthening its applicability in data-intensive and security-critical environments.

#### 5.2.4 Feature dimensionality reduction accuracy.

Effective dimensionality reduction is essential for optimizing computational efficiency while maintaining the integrity of discriminative features critical to malware detection

As reported in Table 7, the DBN-GRU model consistently achieved higher dimensionality reduction accuracy across all dataset scales, culminating in 98.6% accuracy at 60,000 data points.In comparison, the best-performing baseline, NMLA-AMDCEF, reached a maximum of 94.0%, whereas MalVulDroid and LinRegDroid peaked at 93.6% and 93.8%, respectively.

**Fig 9:**
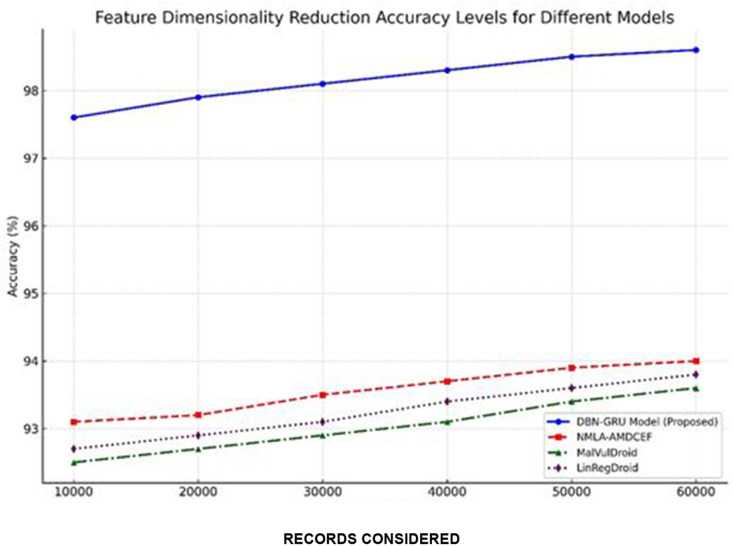
Feature Dimensionality Reduction Accuracy Levels.

The results illustrated in Fig 9 underscore the ability of the DBN-GRU model to retain essential feature representations even after dimensionality reduction, ensuring minimal loss of predictive information.This effective balance between reducing feature complexity and preserving feature relevance further reinforces the model’s suitability for scalable and real-time Android malware detection [40].

#### 5.2.5 DBN-GRU feature processing accuracy.

Feature processing accuracy reflects the model’s ability to transform raw input data into structured, high-quality representations that are suitable for reliable classification.

As presented in Table 8, the DBN-GRU model consistently outperformed the traditional baselines across all evaluated dataset sizes, achieving a peak accuracy of 98.8% at 60,000 records. In comparison, NMLA-AMDCEF, MalVulDroid, and LinRegDroid achieved lower accuracies of 93.3%, 92.7%, and 94.6%, respectively. This consistent performance advantage indicates the robustness of the model in managing complex feature sets while preserving their discriminative utility.

**Fig 10:**
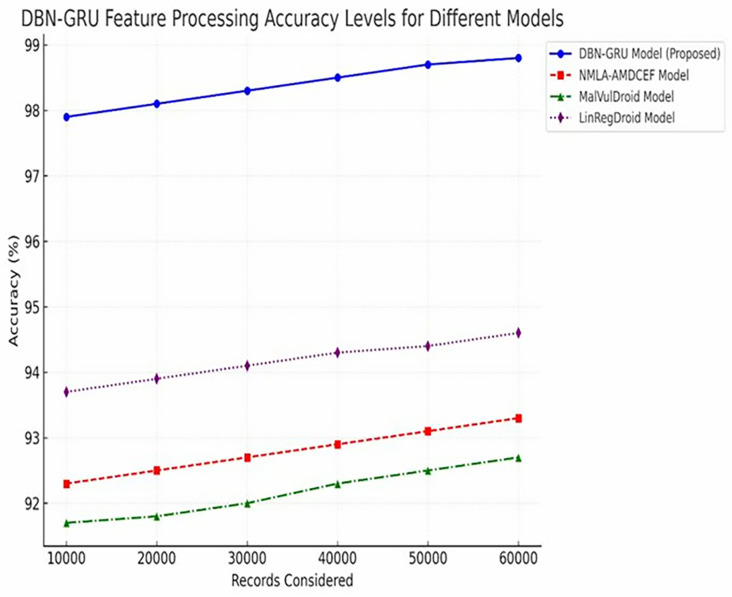
DBN-GRU Feature Processing Accuracy Levels.

Fig 10 further illustrates this trend, confirming the efficiency and precision of the DBN-GRU model in end-to-end feature handling, which is critical for maintaining high detection fidelity in Android malware classification tasks.

#### 5.2.6 Malware detection time analysis.

Rapid detection is a critical requirement for real-world malware mitigation, where latency directly affects the effectiveness of threat responses.

As shown in Table 9, the DBN-GRU model consistently achieved lower detection times across all dataset sizes, completing malware identification in only 16.2 s for 10,000 records and scaling to 17.0 s for 60,000 records. In contrast, baseline models such as NMLA-AMDCEF, MalVulDroid, and LinRegDroid exhibited significantly higher detection latencies, with MalVulDroid requiring up to 25.2 s.

**Fig 11:**
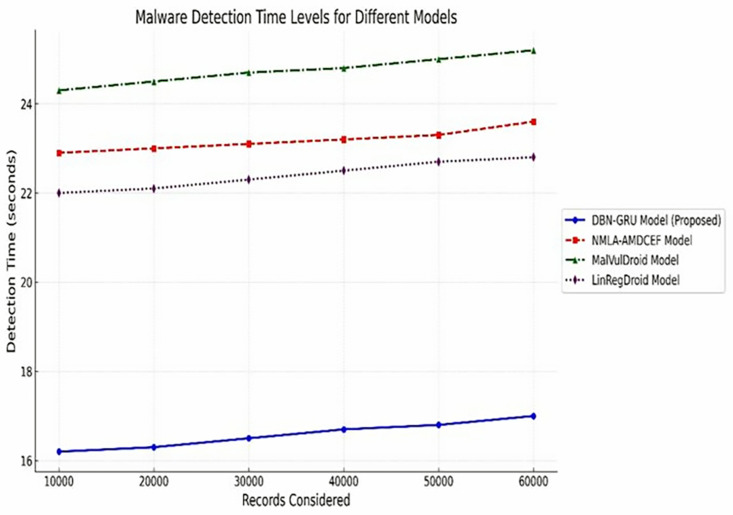
Malware Detection Time Levels.

This performance gap, visually reinforced in Fig 11, demonstrates the computational efficiency and responsiveness of the DBN–GRU model.Its optimized detection pipeline enhances its applicability in time-sensitive environments, where swift and accurate classification is essential for real-time Android malware protection.

#### 5.2.7 Malicious signal detection accuracy.

Accurate identification of malicious signals is a fundamental requirement for any malware detection system, as it directly influences the model’s ability to generalize across diverse and evolving threat environments.

Table 10 presents a comparative evaluation of the DBN-GRU model against established baselines, NMLA-AMDCEF, MalVulDroid, and LinRegDroid, across varying dataset sizes.The DBN-GRU consistently achieved the highest detection accuracy, starting at 97.9% for 10,000 records and scaling up to 98.7% at 60,000 records.In contrast, traditional models exhibited lower performance ceilings, with LinRegDroid, MalVulDroid, and NMLA-AMDCEF peaking at 95.1%, 94.6%, and 93.2%, respectively.

**Table 10:** Malicious signal detection accuracy levels.

Records Considered	DBN-GRU Model (%)	NMLA-AMDCEF (%) [12]	MalVulDroid (%) [13]	LinRegDroid (%) [14]
10,000	97.9	92.2	93.7	94.1
20,000	98.0	92.5	93.8	94.3
30,000	98.1	92.7	94.0	94.6
40,000	98.3	92.8	94.1	94.8
50,000	98.5	93.0	94.4	95.0
60,000	98.7	93.2	94.6	95.1

**Fig 12:**
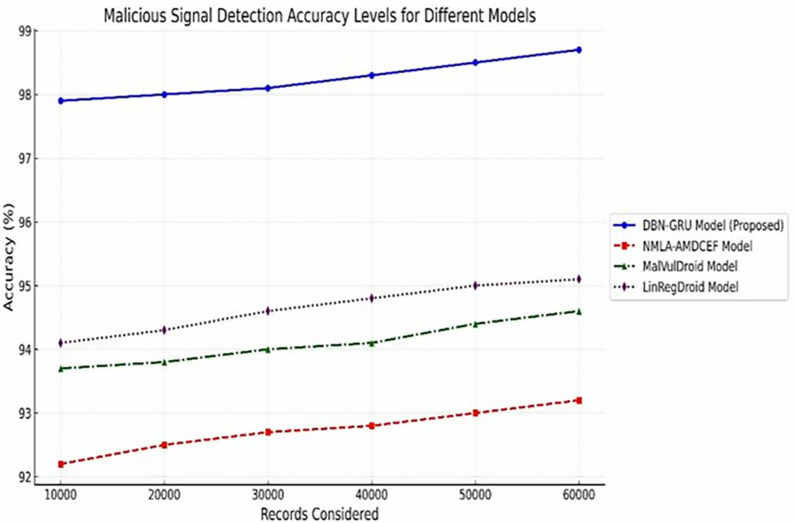
Malicious Signal Detection Accuracy Levels.

As shown in Fig 12, the consistent superiority of the DBN–GRU model reflects its ability to robustly identify subtle and obfuscated malicious patterns, making it particularly effective for large-scale, real-time Android malware detection.These findings reinforce the model’s readiness for deployment in dynamic environments, where precision and adaptability are paramount.

### 5.3 Adaptability and generalization capability

The proposed model consistently outperforms traditional approaches across multiple performance metrics, demonstrating strong detection capabilities against both known and obfuscated malware patterns.In addition to its performance, the proposed DBN-GRU model demonstrates strong adaptability to evolving malware threats.This is attributed to the model’s ability to generalize across new and previously unseen malware variants by learning the deep feature abstractions.The GRU component effectively handles variable-length input sequences, enabling the detection of diverse behavioral patterns over a period.Furthermore, the architecture is well-suited for continual learning, allowing it to be retrained on updated datasets without requiring a structural redesign.These qualities collectively enhance the resilience and long-term applicability of the model in dynamic threat landscapes.

### 5.4. Future directions and limitations

Although the DBN-GRU model demonstrates substantial promise in Android malware detection, several areas remain open for further refinement and exploration.Enhancing its suitability for real-time deployment will require targeted efforts to minimize computational latency and improve responsiveness under constrained operating conditions.Although the current evaluation is based on the Drebin dataset, future studies must incorporate diverse and contemporary datasets to better reflect the evolving nature of malware and assess the generalizability of the model across broader threat landscapes.

Another critical avenue is strengthening resilience against adversarial attacks and evasion strategies.Employing adversarial training techniques and robust regularization can enhance the model’s capacity to withstand such obfuscation and manipulation. In parallel, the integration of Explainable AI (XAI) frameworks can support interpretability and transparency, which are vital for trust and accountability in high-stakes security environments.

Adaptation to resource-constrained platforms, such as mobile and embedded systems, will benefit from the implementation of model compression, pruning, and lightweight architectures.Additionally, the inclusion of multimodal data sources, such as real-time network traffic and system logs, could further enrich the detection pipeline and increase threat coverage.

Despite its high accuracy and processing efficiency, the performance of the DBN-GRU model under extreme data scales and heterogeneity requires ongoing optimization.Addressing overfitting through expanded training sets and refined regularization strategies is essential to maintain performance on unseen data. Collectively, these future directions underscore the importance of continuous innovation to ensure that the DBN-GRU framework remains adaptable, efficient, and robust in the face of increasingly sophisticated mobile malware threats.

## 6. Conclusion

This study introduces a hybrid DBN-GRU model for Android malware detection that strategically integrates static and dynamic analyses to address the limitations inherent in conventional detection frameworks.By leveraging the hierarchical feature extraction capabilities of Deep Belief Networks (DBNs) alongside the temporal modeling strength of Gated Recurrent Units (GRUs), the proposed architecture demonstrates marked improvements over established baselines—namely NMLA-AMDCEF, MalVulDroid, and LinRegDroid—across critical performance metrics, including accuracy, precision, recall, and AUC.

Empirical evaluations confirm the model’s proficiency in identifying both known and obfuscated malware patterns with high reliability and minimal latency, positioning it as a robust solution for contemporary mobile-security challenges.Its ability to generalize complex behavioral patterns reinforces its practical applicability in dynamic threat environments.In the future, extending the model’s capabilities through adversarial robustness techniques, ontology-driven feature enrichment, and architectural scaling will be essential for supporting real-time deployment.Incorporating explainability mechanisms further enhances trust and operational transparency. Overall, the DBN-GRU framework offers a scalable and resilient foundation for next-generation malware detection systems, paving the way for future advancements in large-scale, real-world cybersecurity applications.
